# Bodily Deformities and Their Treatment

**Published:** 1885-09

**Authors:** 


*Bodily Deformities and their Treatment.* By H. A. REEVES. Pp. 460. London: H. K. LEWIS. 1885.

The principle of special men for special subjects, a homogeneous series and one publisher, is a novel feature of medical book production. Wood & Co. have had their series; Cassell & Co. have their series already well advanced; Lewis has had his series in progress for a longer period, but its growth is not very rapid. So far, however, it is good. The author of the work before us appears to be a many-sided man. In the broadest sense, he appears to be a general surgeon; and in the most liberal possible sense, he is at the same time a specialist. We fully admit the capacity of such a man to instruct us in subjects of the greatest breadth, as well as of the most rigid circumscription; and in the work before us we see no reason to depart in practice from the liberality which we would grant him in theory.

Mr. Reeves's work is certainly a good one. It has all the breadth of the general surgeon, with very little of the narrowness of the specialist. If we wished to be hyper-critical, we should say that it had too much of the freedom of the scribe, with too little of the gravity of the scientist. Discounting the evident facility of penmanship, and balancing this with the evident familiarity with his subject, we must admit that on the whole Mr. Reeves has produced a work bearing evidences of great ability in the handling of theory, and of considerable discrimination in the working out of practice.

There is some originality in the work. The subjects of Spring Finger and Paralytic Dislocations are essentially novel. An ingenious operation for nasal depression is also new to British surgery. His classification of Club-foot is a novelty that is valuable. Most of the illustrations are original, and this is a matter for praise; unfortunately, most of them are bad, and many are simply execrable. We must admit that if we can eliminate the artistic, we can detect in most of them the useful; but surely, in the present day, it is possible to combine both.

If the vices of the book are numerous, they are, probably, unimportant; the virtues are, undoubtedly, numerous and great. The breadth and grasp of the work, the clearness of its language, the precision of its sub-divisions and definitions and classifications, alone make it worthy of theoretical mark and practical value.

